# Nutraceutical Supplementation as a Potential Non-Drug Treatment for Fibromyalgia: Effects on Lipid Profile, Oxidative Status, and Quality of Life

**DOI:** 10.3390/ijms25189935

**Published:** 2024-09-14

**Authors:** Salvador de la Cruz Cazorla, Santos Blanco, Alma Rus, Francisco Javier Molina-Ortega, Esther Ocaña, Raquel Hernández, Francesco Visioli, María Luisa del Moral

**Affiliations:** 1 Department of Experimental Biology, University of Jaén, 23071 Jaén, Spainsblanco@ujaen.es (S.B.); rhernand@ujaen.es (R.H.); mlmoral@ujaen.es (M.L.d.M.); 2 Department of Cell Biology, University of Granada, 18071 Granada, Spain; mrus@ugr.es; 3 Department of Physiotherapy, University of Granada, 18071 Granada, Spain; fjmolina@ugr.es; 4 Unit of Clinic Analyses, Hospital Universitario Ciudad de Jaén, 23071 Jaén, Spain; eocanap@gmail.com; 5 Department of Molecular Medicine, University of Padova, 35121 Padova, Italy

**Keywords:** fibromyalgia, olive oil, proteomics, lipid profile, inflammation, oxidative stress, polyphenols

## Abstract

Fibromyalgia (FM) is a chronic syndrome of unknown etiology, although many studies point to inflammation, oxidative stress, and altered mitochondrial metabolism as some of the cornerstones of this disease. Despite its socioeconomic importance and due to the difficulties in diagnosis, there are no effective treatments. However, the use of non-drug treatments is increasingly becoming a recommended strategy. In this context, the effects of supplementation of FM patients with an olive (poly)phenol, vitamin C, and vitamin B preparation were investigated in this work, analyzing complete blood count, biochemical, lipid, and coagulation profiles, and inflammation and oxidation status in blood samples. To gain a better understanding of the molecular mechanisms and pathways involved in the etiology of FM, a proteomic study was also performed to investigate the mechanisms of action of the supplement. Our results show that the nutraceutical lowers the lipid profile, namely cholesterol, and improves the oxidative status of patients as well as their quality of life, suggesting that this product could be beneficial in the co-treatment of FM. ClinicalTrials.gov (ID: NCT06348537).

## 1. Introduction

Fibromyalgia (FM) is a complex disease characterized by chronic widespread pain accompanied by a bad night’s sleep, generalized fatigue, and cognitive problems, among others [1].

The etiology of FM is still poorly elucidated and there is insufficient evidence to identify the specific cause for the development of this disease [1]. In fact, it is currently believed that multiple factors influence its progress, including dysfunctions in the nervous system [2] and genetic [3,4], lipid [5,6], hormonal [7], or immunological/autoimmunological alterations [8] that may be related to both the inflammatory [9] and oxidative statuses underlying this disease [10].

The lack of consensus on the etiopathogenesis of FM makes this illness difficult to diagnose. In this sense, the diagnosis is mainly based on clinical data [11]. Thus, a series of questionnaires have been designed and incorporated into practice, such as the FIQR (Revised Fibromyalgia Impact Questionnaire, [12]) and the SF-12 (Short Form Health Survey) [13]. They are especially useful in the assessment of the impact of FM and in the determination of the physical and psychological quality of life of patients diagnosed with FM, respectively.

At present, there are no specific laboratory tests that can confirm the diagnosis of FM [14]. Nevertheless, different studies in biological fluids have analyzed inflammatory [15], lipid [16], and oxidative [17] parameters in the search for potential biomarkers for FM. However, these works have not yielded specific or exclusive biomarkers. In addition, several approaches based on proteomic studies are being carried out to detect a specific signature in this pathology. Hence, different studies have analyzed the proteome of patients with FM in saliva [18], serum [19], cerebrospinal fluid [20], and urine [21]. One of the most used techniques in these studies is liquid nano-chromatography coupled to label-free tandem mass spectrometry (label-free nLC-MS/MS). Using this approach, 33 differentially expressed proteins were found in the plasma of FM patients [22], mostly related to the inflammatory state, e.g., haptoglobin and fibrinogen [23].

Presently, there is no effective drug treatment for FM; as an alternative, the use of non-drug treatments for FM is widely recommended and accepted [1,24,25]. Accordingly, the combination of pharmacological therapies with non-pharmacological treatments appears as a promising approach [26]. Following this hypothesis, nutritional interventions appear to offer auspicious results in the management of FM [27]; nevertheless, these studies are preliminary and not yet conclusive [28]. In this context, Martínez-Rodríguez and collaborators [29] have suggested that following a Mediterranean Diet (MD) may entail an interesting strategy for FM patients. The administration of the MD together with magnesium and tryptophan supplementation has shown significant improvements in fatigue, anxiety, and depression in those patients.

Among the nutraceutical components that are gaining traction, olive (poly)phenols stand out because of their manifold biological actions [30]. Indeed, (poly)phenolic compounds are mostly responsible for the beneficial properties of extra virgin olive oil (EVOO) and several studies have analyzed their pharma-nutritional properties [31]. With regard to FM, Rus et al. carried out a nutritional intervention with three types of olive oil that differed in their composition of (poly)phenolic components. Only the FM patients who were administered EVOO improved the quality of life and the oxidative status [32] The proposed mechanisms of action include the anti-inflammatory actions of olive (poly)phenols such as hydroxytyrosol (HT) and oleocanthal [30,31] as well as their augmentation of mitochondrial activity [30,33], which in turn may contribute to the process of nociception and widespread pain characteristic of FM [34]. Therefore, the administration of HT and other olive (poly)phenols might help partially resolve FM.

Ascorbic acid is a circulating antioxidant [35,36] endowed with vasodilating properties mediated by enhanced nitric oxide production [36]. Unfortunately, a considerable and expectedly increasing proportion of the world population is unable to achieve an adequate target plasma concentration with the current recommended daily intake of vitamin C [36,37]. With regard to FM patients, the administration of ascorbic acid decreased lipid peroxidation in a pilot trial without significant clinical improvements [38]. In other trials, however, vitamin C did improve symptomatology [24], possibly because of dose or patient selection differences.

B Vitamins are important for cell metabolism and insufficient intake by, e.g., the elderly, vegans, vegetarians, proton pump inhibitors users, etc. [39], and often lead to deficiencies that result in fatigue among other neurological symptoms [40,41].

In light of the above, we undertook a pilot trial of a nutraceutical preparation rich in (poly)phenols in patients with FM. To investigate the mechanisms of action, we have studied the complete blood count (CBC), the biochemical, lipid, and coagulation profiles, and the inflammatory and oxidative statuses in blood samples from these patients. In addition, to achieve a better understanding of the molecular mechanisms underlying this disorder and the effects of the nutraceutical preparation, we carried out a proteomic study in the plasma samples of these patients. With the aim of broadening the scope of the study and obtaining a global view, we have also assessed the clinical conditions of the individuals.

## 2. Results

### 2.1. CBC, General Biochemistry, Lipid Profile, Inflammatory and Oxidative Statuses, and Clinical Conditions

#### 2.1.1. CBC

No significant differences were found in any of the parameters analyzed when comparing the effect of the administration of placebo and Mygrium^®^ to FM patients (Table 1).

#### 2.1.2. General Biochemistry

**Figure 1:**
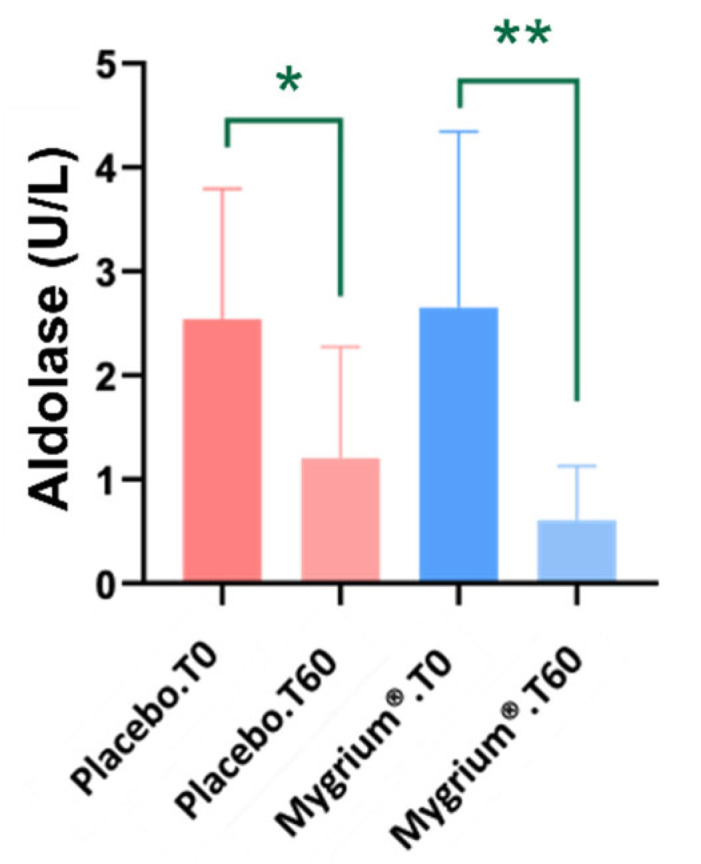
A significant decrease in aldolase concentration was observed in both groups, being greater in the patients treated with Mygrium^®^. * *p* ≤ 0.05; ** *p* ≤ 0.01.

Aldolase concentrations decreased significantly in both the placebo and Mygrium^®^ groups, although the decrease was more pronounced in patients treated with Mygrium^®^ (Figure 1). Increases in aldolase levels have been detected in myotonic muscle disease [42]. At present, aldolase is not widely used as a biomarker of skeletal muscle injury, but it is conceivable that a decrease in aldolase activity is partially responsible for the improvement in symptomatology, but we cannot exclude reverse causality.

#### 2.1.3. Lipid Profile

**Figure 2:**
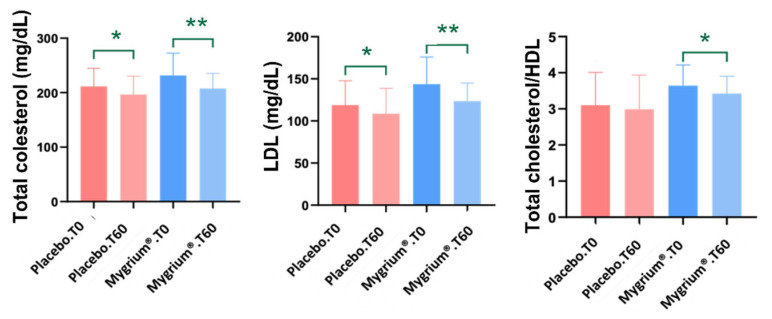
Total cholesterol, LDL cholesterol, and cholesterol ratio data showed a decrease after 60 days of treatment in both groups. * *p* ≤ 0.05; ** *p* ≤ 0.01.

**Figure 3:**
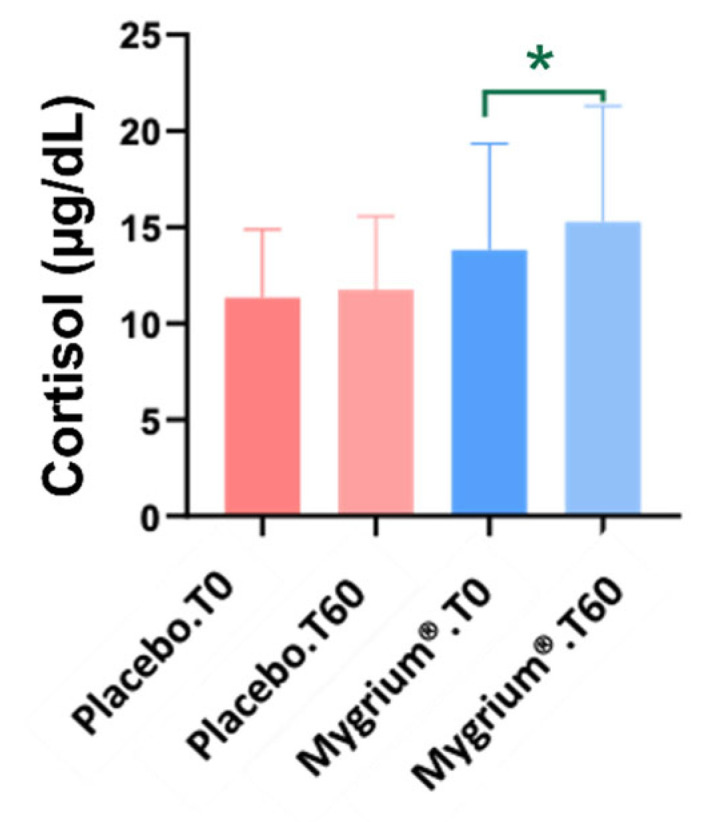
Cortisol values increased significantly only in patients treated with Mygrium^®^. * *p* ≤ 0.05.

Total cholesterol, cholesterol ratio, and LDL cholesterol dropped in both groups, but the decrease was higher in patients treated with Mygrium^®^ (Figure 2). On the other hand, cortisol increased slightly but significantly in the latter group (Figure 3).

#### 2.1.4. Inflammatory and Oxidative Statuses

**Figure 4:**
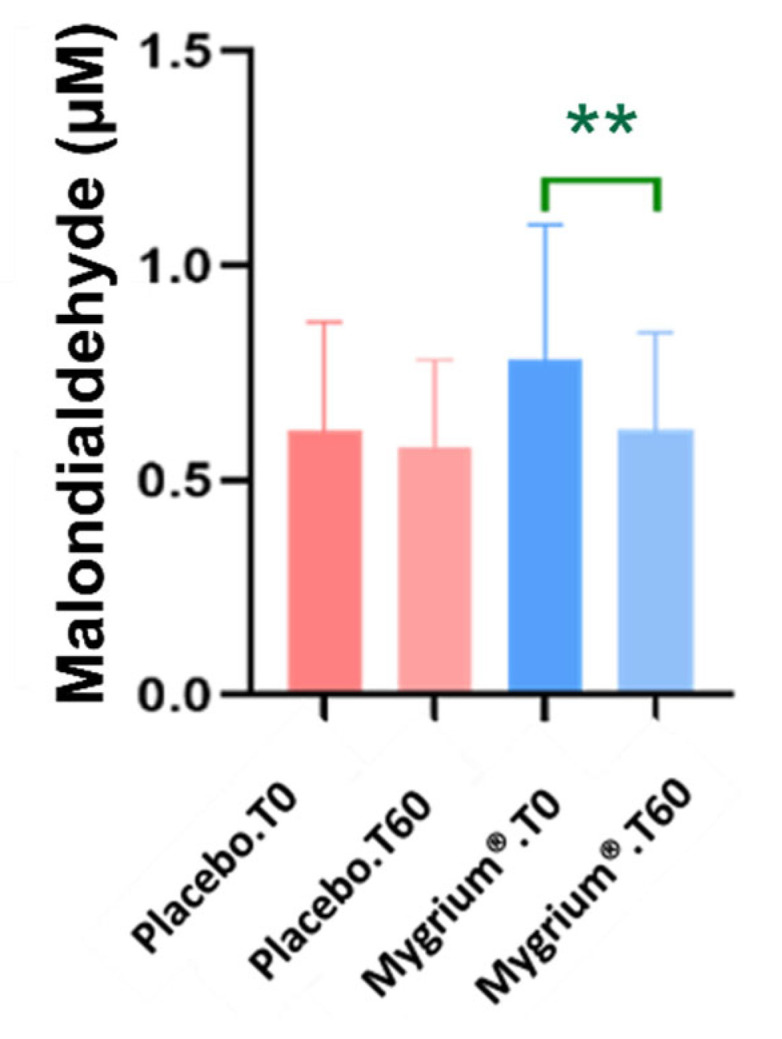
Thiobarbituric acid reactive substances (TBARS) levels decreased significantly in patients administered Mygrium^®^. ** *p* ≤ 0.01.

The nutraceutical significantly decreased lipid peroxidation concentrations (Figure 4).

#### 2.1.5. Clinical Condition: Quality of Life Questionnaire

**Figure 5:**
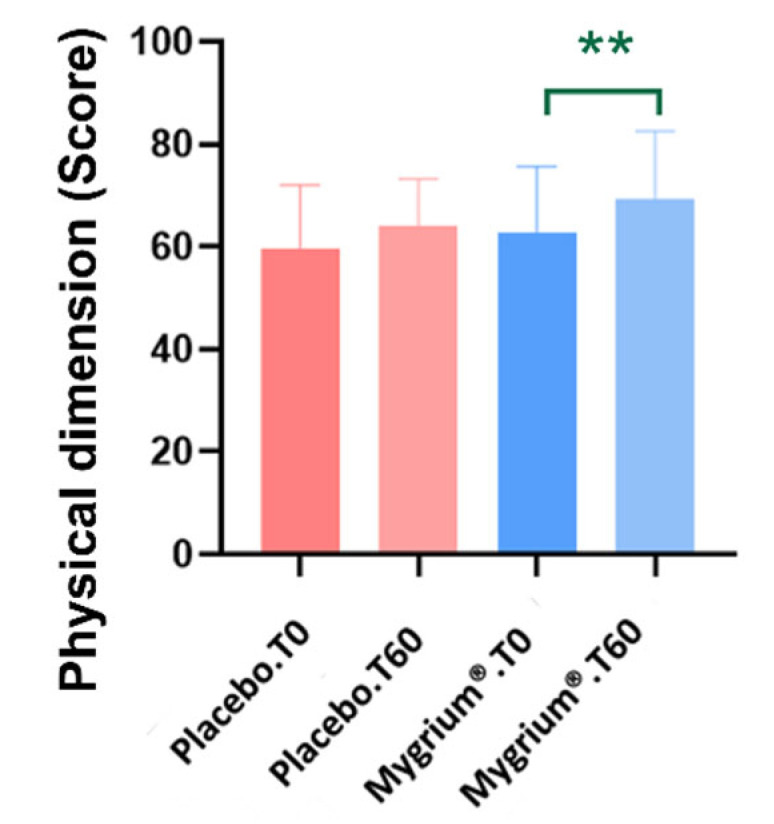
The physical dimension outcomes showed better performance in the group of individuals treated with Mygrium^®^. ** *p* ≤ 0.01.

No significant differences were noted on either the psychic dimension or FIQR. Yet, patients under treatment with Mygrium^®^ experienced better outcomes on the physical dimension (Figure 5).

### 2.2. Plasma Proteome

The bioinformatics analyses were performed using the KEGG (https://www.genome.jp/kegg/), STRING (https://string-db.org), and Top Gene (https://toppgene.cchmc.org) platforms.

1. KEGG’s Mapper tool helped to find out the molecular pathways in which proteins are involved, highlighting the immune response (complement and coagulation cascade, platelet activation, etc.), diseases that produce inflammation (COVID-19, systemic lupus erythematosus, etc.), and cholesterol metabolism (Table 1);
2. Using STRING, the types of interactions between the proteins introduced were evaluated. We also obtained information about the biological processes in which they were involved, highlighting, as with KEGG, different pathways related to the immune response, such as the complement cascade and platelet degranulation (Table 1). Using the STRING tool, the different protein–protein interactions between the proteins detected in the proteome obtained at T60 and T0 of patients treated with placebo and Mygrium^®^ can be evaluated. Regarding the comparison at T60 of the proteome of the two groups of patients, only two proteins were differentially expressed, making it unfeasible to perform this type of analysis. On the other hand, the proteins differentially expressed after treatment with Mygrium^®^ are related to the structure of the exosomes;
3. Finally, the Top Gene tool allowed us to carry out an enrichment analysis based both on the functional annotations of the genes introduced and on the interactions of the problem proteins. In this case, the proteins identified were also involved in the immune response and in cholesterol metabolism (Table 1).

**Figure 6:**
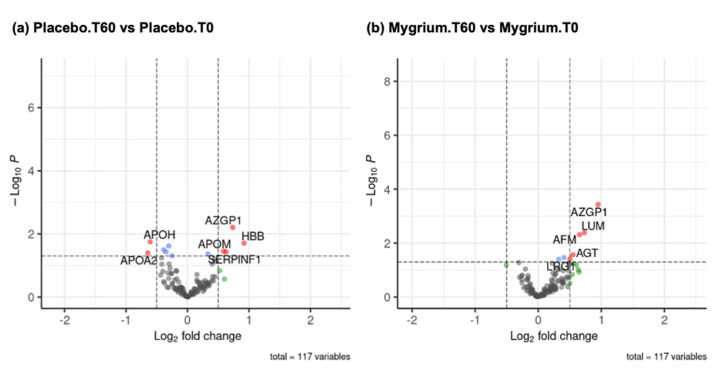
Volcanoes of the comparisons of proteins detected between the different groups of patients and between different times (*p*-value ≤ 0.05; fold change ≥ 1). The points in the center of these volcanoes correspond to those proteins that do not vary in the two situations foreseen in our experimental design. The proteins with statistically significant differences are presented at the right (overexpressed or upregulated) and left (less expressed or downregulated) of the graph.

As mentioned, not all the proteins detected were differentially expressed between the different groups of patients and between different times. Figure 6 shows two volcanoes for the two comparisons, identifying those proteins that are above a very strict threshold. Table 2 shows the proteins differentially expressed in each comparison and depicted in each volcano, together with the calculated *p*-value and fold change.

## 3. Discussion

Despite the number of attempts to find an efficient treatment for FM, no effective molecule has been found yet. In fact, the studies carried out in recent years suggest non-pharmacological therapies, alone or in combination with pharmacological treatments, as a promising approach.

Considering this background, and given the inflammatory and oxidative bases underlying this disease, we have assessed the effect of the administration of a novel preparation in patients suffering from FM.

### 3.1. CBC, General Biochemistry, Lipid Profile, Cortisol Inflammatory and Oxidative Statuses, and Clinical Conditions

#### 3.1.1. CBC

Regarding CBC, we found no statistical differences in any of the parameters analyzed, including red blood cell count, hematocrit, mean corpuscular hemoglobin, or medium corpuscular volume. Interestingly, these parameters have been previously described to be altered in patients with FM [43]. In addition, these variables are closely related to anemia, a pathology commonly described as concomitant with FM [44], where lower levels of hemoglobin were observed in patients with FM [45]. Furthermore, recent studies have pointed to anemia as a risk factor for FM [46]. These discrepancies call for establishing more specific clinical parameters for the diagnosis of FM.

#### 3.1.2. General Biochemistry

Clinical blood biochemistry shows no variations in the parameters analyzed, except for aldolase. Of note, aldolase activity can be used to assess the state of muscle mass [47] and FM progression [18]. With regard to the latter, potential interpretation involves the mitochondrial dysfunction noted above as a possible etiological agent of fibromyalgia [5,48]. To balance the energetic problem derived from mitochondrial dysfunction, anaerobic metabolism is activated with an increase in aldolase activity [19]. Furthermore, this adaptation causes an increase in reactive oxygen species (ROS), which contribute to nociception and generalized pain [49,50]. In this study, a significant decrease in aldolase activity has been reported with both treatments, with this decline being greater in the patients treated with Mygrium^®^. This result suggests that Mygrium^®^ produces some improvement in mitochondrial activity and in oxidative status through lower ROS production.

#### 3.1.3. Lipid Profile

Some studies point to an association between hyperlipidemia, risk of cardiovascular disease, and FM [5,6,51,52]. In agreement with those pieces of work, our results show that the mean basal serum total cholesterol was higher than the reference values in both groups (placebo and Mygrium^®^). Nevertheless, after two months of treatment, this value decreased in both groups of patients. However, the decrease was only significant in the group of patients treated with the nutraceutical preparation, and so was the LDL/HDL cholesterol ratio.

Regarding the low-density lipoprotein cholesterol (LDL-c), it is well established that its atherogenic properties are in part due to the modifications in size and composition that this protein undergoes when the apolipoprotein B (apoB) loses sialic acid residues (desaalylation) [53,54]. Apolipoproteins are not only involved in the maintenance of lipoprotein structure, but they also participate in lipoprotein metabolism by activating and inhibiting enzymes and transferring lipids between lipoproteins. Specifically, apoB promotes the accumulation of cholesterol in the atheromatous plaque [55] and its plasma value is related to the risk of cardiovascular disease [56]. Our data show that both the placebo and the nutraceutical preparation decreased the LDL-c and apoB values, but the decrease was only significant after treatment with Mygrium^®^.

Epidemiological studies have investigated the beneficial health effects of olive biophenols and their pharma-nutritional properties [30,57]. Some studies report that these compounds have cardioprotective activities [31] and also improve the lipidic profile [58,59], but there is no consensus on this issue [31,60,61]. Our data show that the administration of an olive (poly)phenol to patients suffering from FM augments their lipid profiles, thereby decreasing their cardiovascular disease risk and adding to the debate. Also, a recent trial with vitamin C (another component of Mygrium^®^) reported mild improvements in metabolic parameters [62]. Synergistic effects can perhaps explain our data, but proper trials are necessary to confirm this hypothesis.

#### 3.1.4. Cortisol

Chronic pain, the main symptom of FM, is related to cortisol levels, via some unclear mechanisms [63]. Some studies point to an inversely proportional relationship between perceived pain and cortisol levels [64,65]. Conversely, other works indicate a monotonic association between these two parameters [66]. Here, we report a significant increase in cortisol levels after the administration of the nutraceutical, which might be involved in the improved pain perception reported by patients [67].

#### 3.1.5. Inflammatory and Oxidative Statuses

In FM (and beyond), inflammation and oxidative stress are closely related [68]. In fact, oxidative stress does likely play a major role in neuroinflammation associated with FM [69]. More specifically, the mitochondrial dysfunction associated with inflammatory diseases usually leads to an increase in ROS production [70,71]. Moreover, ROS can cause further inflammation, creating a vicious cycle [72,73]. The elevated levels of ROS found in FM patients [5,74] can easily lead to enhanced lipid peroxidation [75]. Interestingly, we found a clear and significant decrease in lipid peroxidation, assessed by the TBARS method, in the patients treated with Mygrium^®^, suggesting that olive (poly)phenols and ascorbic acid ameliorate oxidative stress in FM patients. At a molecular level, Ramirez-Tejero et al. carried out a study on dermal fibroblasts from healthy and FM female patients treated with HT [22]. After proteomic analysis, the fibroblasts from the non-treated patients diagnosed with FM showed differential expression of proteins involved in the turnover of the extracellular matrix and in the oxidative metabolism, which could explain the inflamed status of these patients. Interestingly, a number of these proteins were normalized by the treatment with HT [22]. Finally, it is worth noting that olive (poly)phenols can legally claim health actions, precisely on the protection of circulating lipids from oxidation [57].

The rest of the inflammatory/oxidative parameters analyzed, however, showed no significant differences between treated and non-treated patients, calling for further research and in agreement with other publications [51,76].

#### 3.1.6. Clinical Conditions

The revised Fibromyalgia Impact Questionnaire (FIQR) is useful in clinical practice for evaluating the effects of therapies [77]. Here, we report that both Mygrium^®^ and the placebo improved FIQR scores. Yet, the SF-12 questionnaire, which covers both the psychic and psychiatric dimensions of the patients, showed that the patients treated with Mygrium^®^ experienced better outcomes on the physical dimension. In agreement with our results, San Mauro-Martin and collaborators [78] reported an improvement in the quality of life of FM patients following the administration of dietary supplements rich in olive (poly)phenols. Likewise, previous results from our laboratory showed an improvement in the functional capacity of these patients after administration of EVOO (rich in polyphenols) [32,78].

The similar effects of Mygrium^®^ and the placebo may be due to a placebo effect that involves increased resilience, hope for treatment, and optimism during this study, which is commonplace in chronic pain, including FM studies [79,80].

### 3.2. Plasma Proteome

Very few studies analyzed the effects of olive phenols on the tissue proteome, e.g., [81]. To gain some insight into the mechanisms of action of Mygrium^®^, we analyzed the plasma proteome.

Once the differentially expressed proteins were identified in the plasma of patients in both treatment groups, bioinformatics tools were used to analyze the pathways, biological processes, and protein–protein interactions. All the results obtained by means of KEGG, STRING, and Top Gene tools pointed out that the activated pathways were related to coagulation and the complement system. These outcomes are consistent with those previously obtained by our group when comparing the plasma proteome of patients with FM and healthy subjects [22]. These pathways play an important role in inflammation, highlighting the importance of the inflammatory state in the etiology and pathogenesis of FM [82]. Indeed, these routes are overexpressed in other pathologies involving a generalized inflammatory state, such as rheumatoid arthritis [83] and systemic lupus erythematosus [84].

Earlier proteomic results from our laboratory showed the effect of hydroxytyrosol (HT), the foremost phenolic component of virgin olive oil, on dermal fibroblasts from FM patients [85]. We reported that fibroblasts from FM showed a differential expression in proteins involved in the turnover of extracellular matrix (ECM) and oxidative metabolism, which could also explain the inflammatory status of these patients. After HT treatment, a number of those proteins were normalized. This fact seems to support that an HT-enriched diet could be highly beneficial in the management of FM.

In the current study, five upregulated proteins have been detected after the administration of the polyphenol-rich nutraceutical preparation.

i. Zinc-alpha-2-glucoprotein (AZGP1, a secretory glycoprotein of ~40 kDa) stimulates lipid degradation in adipocytes. The adipose tissue is one of the most involved in metabolism. It has been described that HT supplementation differentially affects the adipose and liver tissue proteome [81]. Moreover, studies have shown that blood AZGP1 is significantly increased in patients with chronic hemodialysis and early acute kidney injury [86,87]. Therefore, the observed increase in AZGP1 might be beneficial on one side yet noxious on the other one. Speculatively, the overall balance favors symptom improvement.
ii. Afamin (AFM) is a protein involved in the transport of hydrophobic molecules such as lipids and vitamins. It is a human vitamin E binding glycoprotein and is a member of the human albumin gene family including albumin, alpha-fetoprotein, and vitamin D–binding protein; it is mainly expressed in the liver and secreted into the bloodstream, with a molecular mass of 87,000 Dalton [88]. Its biological actions are still quite vague and in need of deep investigation [88].
iii. Leucine-rich alpha-2-glycoprotein 1 (LRG1) is linked to the transformation of the growth factor receptor beta. Research on LRG1 and its involvement in the occurrence and development of diseases was still in its infancy and studies are now focused on proteomic detection and basic animal experimental reports [89].
iv. Lumican (LUM), a proteoglycan of the extracellular matrix, mediates collagen binding. We had previously unveiled that fibroblasts from FM showed a differential expression in proteins involved in the turnover of the extracellular matrix [85]. Also, lumican is a structure regulatory proteoglycan of collagen-rich tissues, with cell instructive properties through interactions with a number of cell surface receptors in tissue repair, thereby regulating cell proliferation, differentiation, inflammation, and the innate and humoral immune systems to combat infection [90].
v. Angiotensinogen (AGT), an essential component of the renin–angiotensin system, regulates blood pressure, blood flow, and electrolyte homeostasis.

These five differentially expressed proteins might be related to exosomes, extracellular vesicles secreted by different cells that transport various substances [91]. Of note, these particles have been linked to different pain-related pathologies [92]. As the field of exosomes/nanovescicles is receiving considerable attention, it would be interesting to study the role of exosomes in FM because they may be involved in the inflammatory state and chronic pain perception, two of the main features of FM [93].

The net sum of the biological effects of the above proteins is difficult to determine but confirms that the effects of some components of Mygrium^®^ such as olive (poly)phenols could be mediated by the proteome [81]. At this stage, these data should be interpreted with caution and in a descriptive manner. The main limitations of this study include the small sample size (this study should be considered a pilot study) and the lack of complete elimination of symptoms. Nutraceuticals alone will not cure fibromyalgia, but they may be useful in alleviating some components of symptomatology. Future studies of longer duration and with a larger sample size will extend the results and provide more data on the use of bioactive molecules in this debilitating condition.

In conclusion, Mygrium^®^ improves some of the features of FM, notably the mental quality of life and lipid profilevia of anti-inflammatory, antioxidant, and proteome-modulating actions. Further studies with different doses and larger cohorts will eventually provide more data to back the potential use of this nutraceutical in the adjunct treatment of fibromyalgia.

## 4. Materials and Methods

### 4.1. Experimental Design and Treatments

A randomized double-blind placebo-controlled nutritional intervention has been designed with the nutritional supplement Mygrium^®^ (Solvitae Medical, s.l. Madrid, Spain). Mygrium^®^ is an olive extract standardized in phenolic compounds whose composition per capsule is olive extract (including, among others, Oleacore^®^, Olivenova, 29640, Malaga, Spain): 300 mg (total polyphenols: 42.5 mg, containing hydroxytyrosol, tyrosol, oleuropein, caffeic acid, luteolin and verbascosides); Vitamin C: 60 mg (75% VRN); Niacin: 16 mg (100% VRN); Pantothenic acid (B5): 6 mg (100% VRN); Riboflavin (B2): 1.4 mg (100%); Pyridoxine (B6): 1.4 mg (100% VRN); and Thiamin (B1): 1.1 mg (100% VRN).

### 4.2. Study Population

All patients included in the study were females over 18 years of age who belonged to the FM Association of Jaén “Afixa”. The patients had been previously diagnosed with FM according to the 1990 criteria of the American College of Rheumatology and their participation in this study was voluntary. All patients provided informed consent.

The exclusion criteria were the following:

- Other chronic diseases, namely diabetes mellitus, hypertension, cardiovascular disorders, and cancer;
- Patients with grade II obesity (body mass index ≥ 35 kg/m^2^);
- Active smokers;
- Pregnancy;
- Regular treatment with acetylsalicylic acid, estrogen, corticosteroids, antioxidants, or lipid-lowering drugs
- Participation in another research study;
- The selected patients were divided into two intervention groups:
- Group 1, consisting of 20 patients diagnosed with FM, received a daily dose of three capsules (two in the morning and one in the evening) of Mygrium^®^ for 60 days;
- Group 2, 20 patients diagnosed with FM, received a daily dose of three capsules (two in the morning and one in the evening) of a placebo (maltodextrins) for 60 days. This intervention group served as a control.

### 4.3. Bioethics

The study was carried out in strict compliance with the provisions of Law 14/2007 on Biomedical Research and following the precepts included in the Belmont Report and the Declaration of Helsinki (updated by the Brazilian Assembly in 2013) for biomedical research. We have also considered the Law on Patient Autonomy 41/2002.

Candidates for participation in this project were interviewed through a Patient Information Sheet. All participants in the study gave informed consent to the use of their samples.

This study was approved by the Research Ethics Committee of the Province of Jaén and the University of Jaén and was registered in ClinicalTrials.gov (ID: NCT06348537). We follow the CONSORT reporting guidelines.

### 4.4. Clinical and Experimental Procedures

To assess the effects achieved with this intervention, a series of variables were analyzed and/or collected both at the beginning (T0) and at the end of the intervention (T60).

1. FIQR (Fibromyalgia Impact Questionnaire), to evaluate the functional capacity in daily living activities [94];
2. SF-12 (Quality of Life Questionnaire). This questionnaire assesses both the physical quality of life (Physical component, PCS-12) and the mental quality of life (Mental component, MC-12) [95];
3. Complete blood count (CBC) and general biochemistry;
4. Coagulation, lipid profile, and oxidative status;
5. Plasma proteome.

The same practitioner carried out all the measurements and tests throughout the study. The questionnaires were completed immediately after blood was drawn. 

#### 4.4.1. Blood Collection and Preparation of Blood Samples

Whole blood samples from patients were collected by venipuncture from the antecubital vein early in the morning to avoid daily variations in the parameters and after 12 h of fasting. Blood was drawn in EDTA tubes and EDTA-free tubes to obtain plasma (for proteomic analysis) or serum (for all other determinations), respectively.

#### 4.4.2. Complete Blood Count (CBC)

This analysis was performed at the Clinical Laboratory Management Unit of the University Hospital of Jaén using a DxH 900 Workcell Automated Hematology Solution (Beckman Coulter, Brea, CA, USA).

#### 4.4.3. General Biochemistry

C-reactive protein (CRP) was measured using an AU 5800 analyzer (Beckman Coulter). This analysis was performed at the Clinical Laboratory Management Unit of the University Hospital of Jaén.

#### 4.4.4. Determination of the Lipid Profile

The serum lipid profile was measured by a spectrophotometric procedure using an Olympus AU 5400 analyzer (Beckman Coulter).

The cortisol level was determined in serum samples by a fluorescence polarization immunoassay using an AxSYM (Abbott Laboratories, Abbott Park, IL, USA).

These determinations were carried out at the Clinical Laboratory Management Unit of the University Hospital of Jaén.

#### 4.4.5. Determination of the Inflammatory and Oxidative Status

Thiobarbituric acid reactive substances (TBARS). TBARS are an acknowledged indicator of lipid peroxidation [96]. TBARS levels were determined spectrophotometrically following the manufacturer’s recommendations (#0801192, TBARS Assay Kit, OXITEK, Buffalo, NY, USA). 

This analysis was performed at the Central Research Support Services of the University of Granada. The reliability of all the tests performed was confirmed by reference to the precision, specificity, and sensitivity of each analyzer used.

#### 4.4.6. Proteomic Analyses

Proteomic analyses were performed at the Central Research Support Services of the University of Jaén by liquid nano-chromatography coupled to label-free tandem mass spectrometry (label-free nLC-MS/MS). This technique allows the relative quantification of the abundance of each protein in each sample, making it possible to assess those with differential expression in the different study groups [97]. For this purpose, the samples were depleted by FASP (Filter Sample Assisted Preparation [98]). Peptides were separated with a nanoLCEASY-nLC system connected to an Orbitrap Q Exactive mass spectrometer. The analysis and quantification of the results obtained were carried out using Maxquant software v2.6.4.0.

### 4.5. Statistical Analysis

The normality of the results was assessed using the Graph Pad Prism statistical program (v8.0.2). To study normality, the Kolmogorov–Smirnov test was applied; a 95% confidence interval was used to determine whether the data set follows a normal Gaussian distribution or not. Once normality was assessed, the data obtained for each group of patients were compared considering the two data collection times (T0 and T60). This analysis was performed following different procedures according to the normality of the data.

The protein quantification generated by Maxquant was statistically analyzed using the R-Bioconductor package MSStats (http://msstats.org/), which allows filtering of the initial list of proteins, differentially detecting those that showed more significant changes in their expression due to the effect of Mygrium^®^ or placebo treatment. To represent the proteins with differentially significant expressions, a scatter plot called “volcano” was used. This graph uses the calculated *p* and fold change values. Based on these parameters, it is possible to detect the proteins with the most statistically significant differences in expression.

Once the differentially and significantly expressed proteins were detected, the biological processes modulated by Mygrium^®^ administration were established using the tools KEGG (Kyoto Encyclopedia of Genes and Genomes), STRING (Search tool for the retrieval of interacting genes/proteins), and Top Gene.

## Figures and Tables

**Table 1:** Outcomes of the bioinformatics analyses performed using the KEGG, STRING, and Top Gene platforms.

Tool	Molecular Pathway	Proteins Involved
KEGG	The complement and coagulation cascades	40
	COVID-19	20
	Systemic lupus erythematosus	13
	Staphylococcus aureus infection	12
	Pertussis	10
	Prion diseases	8
	Cholesterol metabolism	8
	Extracellular accumulation of neutrophils	7
	African trypanosomiasis	7
	Platelet activation	6
	Amoebiasis	5
STRING	Complement activation	43
	Regulation of complement activation	35
	Regulation of the humoral immune response	36
	Platelet degranulation	30
	Cytokine-mediated humoral immune response	31
ToppGene	Positive regulation of fibrinolysis	4/4
	Negative regulation of LDL	3/3
	Regulation of complement activation	2/2
	Positive regulation of neurofibrillary assembly	2/2
	Negative regulation of cholesterol amount	2/2

**Table 2:** Proteins differentially expressed in the comparisons between Placebo.T60 vs. Placebo.T0 and Mygrium^®^.T60 vs. Mygrium^®^.T0.

Comparison	Protein	*p*-Value	Fold Change
Placebo.T60 vs. Placebo.T0	AZGP1	5.37 × 10^−3^	0.747
	HBB	1.73 × 10^−2^	0.948
	ApoH	2.03 × 10^−2^	−0.595
	ApoM	3.11 × 10^−2^	0.596
	SERPINF1	3.60 × 10^−2^	0.639
	ApoA2	4.50 × 10^−2^	−0.637
Mygrium^®^.T60 vs. Mygrium^®^.T0	AZGP1	3.69 × 10^−4^	0.947
	AFM	4.67 × 10^−3^	0.65
	LRG1	3.81 × 10^−2^	0.499
	LUM	4.41 × 10^−3^	0.729
	AGT	2.63 × 10^−2^	0.548

## Data Availability

Data is contained within the article.
